# Efficacy of shared decision making on treatment satisfaction for patients with first-admission schizophrenia: study protocol for a randomised controlled trial

**DOI:** 10.1186/1471-244X-14-111

**Published:** 2014-04-14

**Authors:** Mio Ishii, Yasuyuki Okumura, Naoya Sugiyama, Hana Hasegawa, Toshie Noda, Yoshio Hirayasu, Hiroto Ito

**Affiliations:** 1 Department of Psychiatry, Yokohama City University School of Medicine, 3-9 Fukuura, Kanazawa-ku, Yokohama 236-0004, Japan; 2 Numazu Chuo Hospital, 24-1 Nakase-cho, Numazu, Shizuoka 410-8575, Japan; 3 Department of Social Psychiatry, National Center of Neurology and Psychiatry, 4-1-1 Ogawa-Higashi, Kodaira, Tokyo 187-8502, Japan; 4 Research Department, Institute for Health Economics and Policy, Association for Health Economics Research and Social Insurance and Welfare, 1-5-11 Nishishimbashi, Minato-ku, Tokyo 105-0003, Japan

**Keywords:** Schizophrenia, Shared decision making, Patient satisfaction, Randomised controlled trial, Study protocol

## Abstract

**Background:**

Shared decision making is a promising model for patient-centred medicine, resulting in better clinical outcomes overall. In the mental health field, interventions that consider the patient-centred perspective—such as patient quality of life, involvement in the treatment, treatment satisfaction, and working alliance—have increased and better clinical outcomes discovered for patients with schizophrenia. However, few studies have examined the efficacy of shared decision making for schizophrenia treatment. The objective of this study is to evaluate the effect of a shared decision making intervention compared to treatment as usual on patient satisfaction at discharge for first-admission patients with schizophrenia.

**Methods/Design:**

This is a randomised, parallel-group, two-arm, open-label, single-centre study currently being conducted in an acute psychiatric ward of Numazu Chuo Hospital, Japan. We are recruiting patients between 16 and 65 years old who are admitted to the ward with a diagnosis of schizophrenia without prior experience of psychiatric admission. Fifty-eight participants are being randomised into a shared decision making intervention group or a treatment as usual control group in a 1:1 ratio. The intervention program was developed based on a shared decision making model and is presented as a weekly course lasting the duration of the patients’ acute psychiatric ward stay. The primary outcome measure is patient satisfaction at discharge as assessed by the Client Satisfaction Questionnaire. Due to the study’s nature, neither the patient nor staff can be blinded.

**Discussion:**

This is the first randomised controlled trial to evaluate the efficacy of shared decision making for patients with early-treatment-stage schizophrenia. The intervention program in this study is innovative in that it includes both of the patient and staff who are involved in the treatment.

**Trial registration:**

The study has been registered with ClinicalTrials.gov as NCT01869660.

## Background

The healthcare systems in developed countries are evolving from a paternalistic to a patient-centred approach in which patients are more informed and take more active roles in decision making [1,2]. Shared decision making (SDM) is a promising model for the movement in which patients and clinicians share all information and individual preferences regarding the treatment through the entire decision making process [3]. A systematic review of 86 randomised trials found that SDM, when compared to usual care, resulted in patients gaining more knowledge, increased confidence in decisions, and more active patient involvement. These results were found across several fields of medicine, such as cancer treatment [4].

In the mental health field, treatment adherence has remained a common issue over several decades, particularly for patients with schizophrenia. A systematic review found that 49.5% of patients with schizophrenia were estimated to be non-adherent to antipsychotic medication [5]. Numerous interventions have attempted to address this issue, including psychoeducation, motivational and behavioural therapy, family therapy, and telephone call prompts. However, randomised controlled trials and reviews of those interventions have not found enough evidence regarding how to increase adherence, particularly in long-term follow-up studies [6-11]. Therefore, interventions that do not only focus on improvement to adherence but also consider the patient-centred perspective—such as the patient’s quality of life, involvement in the treatment, treatment satisfaction, and working alliance—have increased, thereby resulting in better clinical outcomes for patients with schizophrenia [12-14]. Attempts to find appropriate approaches for better clinical outcomes for patients with schizophrenia are being undertaken and SDM is expected to be one of them.

To the best of our knowledge, there are only three randomised controlled trials examining the efficacy of SDM for schizophrenia treatment. Hamann conducted of them [15,16], comparing SDM intervention with usual care for inpatients with schizophrenia. In the first study, patients (n = 107) in the intervention group were given decision aids that contained information about pharmacological and psychoeducational treatment options. Patients in the intervention group did not differ in their results from the patients in the control group who were given treatment as usual (TAU) in terms of overall treatment satisfaction, but it did improve their treatment knowledge. In the second study (n = 61), patients in the intervention group attended a shared decision making training program consisting of five one-hour group sessions. The intervention yielded higher participation preferences and increased patients’ desire to have greater responsibility in treatment decisions than in the TAU program; this continued to the six-month follow-up. Krieke [17] evaluated an Internet-based decision aid for schizophrenia (n = 250) in an outpatient setting. Patients in the intervention group were offered the opportunity to make use of the Internet-based information and decision tool. This tool was designed to support patients in acquiring an overview of their needs and appropriate treatment options as provided by their mental health care organisation. Patients in the control group were given their usual care. No differences were found between the intervention and control groups regarding perceived involvement in medical decision making.

More studies are needed to determine the efficacy of SDM for schizophrenia treatment. To confirm what former studies have not—namely, reducing patients’ heterogeneity and developing an intervention that targets both patients and staff—we are conducting this study of SDM for first-admission patients with schizophrenia.

### Objectives

The objective of this study is to evaluate the effect of an SDM intervention compared to TAU on patient satisfaction at discharge and treatment continuation six months post-discharge for first-admission patients with schizophrenia.

## Methods/Design

### Trial design

**Figure 1:**
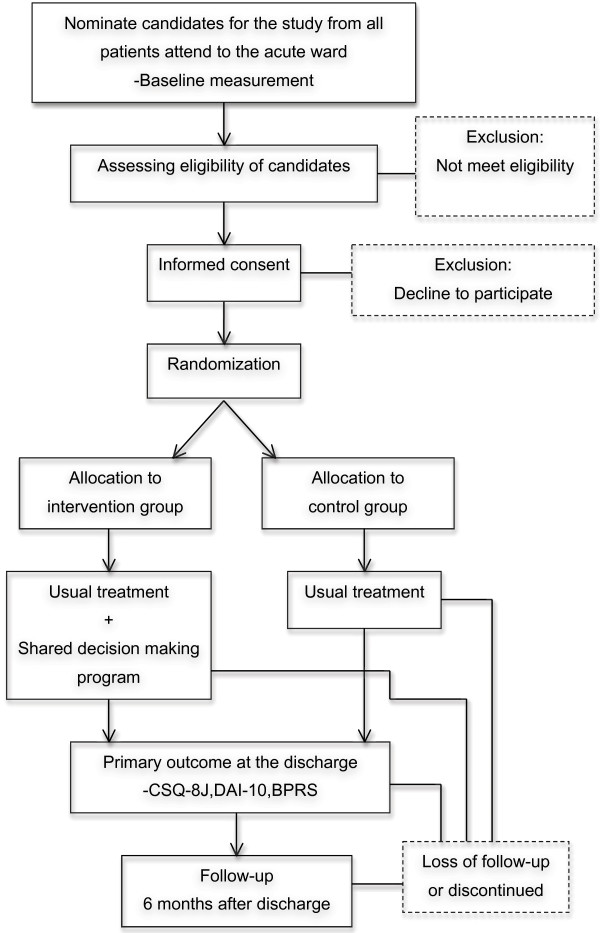
Flow diagram; it provides an overview of the trial flow from baseline assessment at the admission to the follow-up at 6 months after the discharge.

This is a randomised, parallel-group, two-arm, open-label, single-centre study currently being conducted in an acute psychiatric ward in Japan. It has been approved by the Yokohama City University Medical Research Ethics Committee and registered at ClinicalTrials.gov (registration number: NCT01869660). To avoid biased allocation, randomisation is undertaken by central allocation using a computerized random number generator at the Internet Data and Information Center for Medical Research (INDICE), provided by the University Hospital Medical Information Network (UMIN) in Japan. After written informed consent has been obtained, randomisation is performed to assign patients, in a 1:1 allocation ratio, to either the SDM model intervention or TAU with stratification by sex, age (under or over 20 years old), and assumed duration of illness (less or more than one year) via the minimisation method. Due to the nature of the study, neither the patient nor staff can be blinded. Figure 1 provides an overview of the trial flow. Additional file 1 depicts a summary of the study parts and their timing in the intervention and control groups. We estimate that data gathering will be completed in March 2015.

### Study setting

We are conducting our study in an acute psychiatric ward of Numazu Chuo Hospital, a psychiatric hospital with five wards (286 beds in total) in a suburban area of Shizuoka prefecture, Japan. Serving a catchment area of 830,000 people, it is the only psychiatric hospital in the area that accepts emergency admissions 24 hours a day, 365 days a year. On average, approximately 400 people are admitted to the ward in a year and two-thirds of these people are diagnosed with schizophrenia (ICD-10 codes: F20–F29). Approximately 90% of admissions are legally involuntary. The average duration of hospitalisation in the ward is 45 days.

### Eligibility criteria

Participants will be eligible for inclusion in the study if they meet the following criteria: (1) admittance to the acute ward at Numazu Chuo Hospital, (2) schizophrenia diagnosis (ICD-10 codes: F20–F29), (3) aged 16 to 65 years old at admission, and (4) has no prior experience of psychiatric admission (first admission).

Participants are excluded if they (1) are diagnosed with moderate to severe mental retardation, (2) are diagnosed with any of the organic mental disorders (ICD-10 codes: F00–F09), (3) do not have sufficient Japanese language knowledge, or (4) have severe conceptual disorganisation as measured by the Japanese version of the Brief Psychiatric Rating Scale (BPRS; rating of five or more) [18,19]. The cut-off point was decided by referring to prior studies of SDM for inpatients with schizophrenia by Hamann [15,16].

### Intervention

We developed the SDM model program as an inpatient intervention that focuses on the very basics SDM; that is, how patients and medical staff share information and patients’ own preferences regarding the on-going treatment [3].

The program is a weekly course lasting the duration of the patients’ acute psychiatric ward stay, with a maximum of 90 days. Sessions are held on a certain day and time every week during the hospitalisation. They involve three sequential elements: (1) the patient answers the questionnaire regarding their perception of on-going treatment, (2) the patient and staff hold the session in which they share information and their preferences, and (3) the patient and staff create a weekly care plan sheet.

First, participants are asked to complete a six-item self-reported questionnaire that assesses patients’ perceptions of their treatment at the time. The questionnaire (Additional file 2) is an initial intervention tool allowing patients to express themselves more easily and prepare for the following session. Each question is written in a simple sentence, and is designed to be answered using a five-point Likert scale. In order to avoid perceived pressure from staff, the patient is asked to answer in private setting or with the help of a staff member if the patient requests assistance.

Second, to discuss patients’ and medical teams’ perceptions of treatment, patients in the SDM intervention attend a group session. The members of each session are the patient, medical team (i.e. the primary doctor, the primary nurse, and other staff), and a facilitator from the supervision team. Regarding the questionnaire that the patient has answered, the patient and at least three ward staff members discuss for 15–20 minutes the on-going treatment, including medication, ward circumstances, and treatment goals. A facilitator from the study supervision team presides, trying to create a comfortable atmosphere both for the patient and staff members. Other participants are free to discuss their own views and preferences regarding the treatment.

Third, all the session’s participants draft the care plan sheet in order to outline clearly what they have shared in the session. The sheet displays the treatment information at that point in time, including remaining symptoms, diagnosis, the patient’s condition, medication, problems at the ward and solutions, activities, and the goal of hospital treatment. Additional file 3 indicates an example of the care plan sheet.

To standardise and improve adherence to the study, five staff members who are independent from the patient’s treatment are organized into a project supervision team. This team has four duties. First, the team delivers a brief 20-minute lecture regarding the basic theory of SDM to all of the psychiatrists, nurses, social workers, and pharmacologists who work at the ward, prior to their participation in the intervention. Second, the team manages the intervention schedule and facilitates sessions each week. Third, throughout the study period, the team stays in the ward and occasionally provides feedback to participating staff to establish the integrity of the intervention. Fourth, before the weekly intervention session, the team assesses the patient’s condition. If the patient’s condition deteriorates and does not meet the eligibility criteria—for example, conceptual disorganisation worsens to a greater-than-five ranking as per the BPRS—the team postpones the session until the patient recovers.

### Control group

The control group receives TAU, which is mainly medication treatment. During the hospitalisation period, doctors examine patients daily and nurses care for patients by assisting them in their self-care activities and encouraging them to participate in the unit’s daily activity program. Although the primary doctor and nurses discuss the patient’s overall progress and plan for discharge on a regular basis, there is no fixed occasion for the patient and staff to share all of the information. Moreover, there is no specific chance for the patient to be introduced to the concept of SDM or to actively participate in the treatment.

### Primary outcome

This is the patient’s satisfaction at discharge as assessed by the Japanese version of the Client Satisfaction Questionnaire (CSQ-8J) [20,21], a self-report scale containing eight items clustered into four response categories. The overall score is the sum of the item responses; it ranges from 8–32, with higher scores indicating higher satisfaction. The CSQ-8 has been used frequently in clinical trials of schizophrenia [22-24], such as the randomised controlled trials of SDM for schizophrenia by Hamann [15,16]. The Japanese version has good internal consistency (Cronbach’s α = 0.83) and moderate convergent validity (r = .36–.49) with another self-report instrument for client satisfaction inquiry [24] when used for psychiatric inpatients at discharge [21].

### Secondary outcome

These are attitudes toward medication, symptom severity, and treatment continuation. The Japanese version of the Drug Attitude Inventory-10 (DAI-10) [25,26] is used to measure attitudes toward medication. It is a 10-item self-report instrument, with each item being rated as either true or false with regard to the nature of patient experiences with psychotropic drug use. Scores range from -10 to 10, with higher scores indicating a more positive attitude towards medication. The DAI-10 is administered at discharge in the study. The Japanese version of the BPRS, Oxford version, is used to assess symptom severity, which the ward doctor undertakes routinely for all the patients at admission and discharge. Treatment continuation is assessed from their medical records if the patient continues treatment at the Numazu Chuo Hospital and, if not, by asking patients via a telephone call six months post-discharge.

### Sample size calculation

The primary outcome is the mean difference in treatment satisfaction as measured by the CSQ-8J between the SDM group and the TAU group at the time of discharge from the acute psychiatric ward. In the study by Loh et al. [27], among patients with newly diagnosed depression in primary care settings, the standardised mean difference between a SDM intervention group and a TAU group for the CSQ-8 was 0.92. We expect a smaller effect size of 0.80 because the study population of first-admitted patients with schizophrenia is a potentially more complex problem. Assuming a power of 80% and two-sided significance level of 5%, we estimate that a sample size of 26 patients per arm is required to detect an effect size of 0.80. In order to adjust for loss of power due to an anticipated drop out of 10%, 58 patients will be included.

### Statistical methods

The data will be analysed on an intention-to-treat principle. Multiple imputation methods [28] will be used for imputing any missing information regarding outcome measures. Unadjusted comparisons of outcome measures between the groups (SDM vs. TAU) will be conducted using Student’s t test or Mann-Whitney U test, depending on the variable distribution for continuous variables (i.e. treatment satisfaction, attitude toward medication, and symptom severity) and chi-squared test for the categorical variable (i.e. treatment continuation). To control characteristics such as sex, age, and duration of illness, we will use a general linear model or a generalised linear model with log-link function, depending on the variable distribution for the continuous variables and a logistic regression model for a categorical variable. Analyses will be performed using R version 3.0.2.

### Recruitment

The project supervision team checks the medical records of all patients admitted to the acute ward and nominates candidates for the study within three days after admission. After being verified to satisfy all of the inclusion criteria, the patient is asked to give written informed consent by the investigator (MI). The participant or, if the participant is under 20 years of age, the legal guardian, must provide written informed consent before any study procedures occur.

### Research ethics approval

This study is designed and undertaken in compliance with the Helsinki Declaration and the Guidelines on Clinical Research of Japanese Ministry of Health, Labour, and Welfare. The protocol, informed consent forms, and other requested documents, including any subsequent modifications, were reviewed and approved by the Yokohama City University Medical Research Ethics Committee (21/03/2013). The Ethics Committee is monitoring our study and we will withdraw the study immediately on their direction of discontinuation. We will ensure the protection of all participants’ rights during the study. To address the issues associated with obtaining consent from inpatients with schizophrenia who are symptomatic, we exclude individuals who have conceptual disorganisation as measured by the BPRS (rating of five or more). Furthermore, the consent procedure is designed to enhance the intake and retention of information.

## Discussion

This study is the first to evaluate the efficacy of SDM for patients with early-treatment-stage schizophrenia. It is significant that the study targets patients in the early stage of treatment because the effect of psychosocial interventions, such as SDM, would be influenced by past treatment experience and the influence of treatment in the early stages for a long-term outcome is considered strong [12,29]. Another strength of the current study is that the intervention includes both patients and staff. Since SDM is a process based on cooperation between patients and staff, an intervention that includes both sides is needed; however, there were interventions that either focused on the physician’s understanding and communication skills or on informing patients about treatment options [30]. The intervention program in this study is innovative in that it includes the patient and staff who are involved in the treatment, such as the psychiatrist, nurse, social worker, and pharmacist.

There are some difficulties regarding the study design. Performance bias can occur because a blind trial at the intervention level is not possible. The standardised execution of the intervention is another point of concern. To address these issues, a study supervision team that is not involved directly in the treatment observes the protocol and facilitates staff training programs and intervention sessions. Lastly, as the current study is undertaken in a single centre, further research is needed to assess generalizability.

## Abbreviations

SDM: Shared decision making; TAU: Treatment as usual; BPRS: Brief psychiatric rating scale; DAI-10: Drug attitude inventory; CSQ: Client satisfaction questionnaire; INDICE: Internet data and information center for medical research; UMIN: University hospital medical information network.

## Competing interests

The authors declare that they have no competing interests.

## Authors’ contributions

MI is the principal investigator responsible for the initial draft of this manuscript, and for organising and implementing the study. YO calculated the sample size and decided the analytic strategy. YO, NS, and HI contributed to development of the SDM intervention and the study design. NS, TN, and HH contributed to the study’s management. YO also revised earlier versions of the manuscript. All authors read and approved the final manuscript.

## Pre-publication history

The pre-publication history for this paper can be accessed here:

http://www.biomedcentral.com/1471-244X/14/111/prepub

## Supplementary Material

Additional file 1 Parts and timing plot; it is a summary of the study parts and their timing in the intervention and control groups. Click here for file

Additional file 2 Questionnaire for the patient; a six-item self-reported questionnaire that assesses patients’ perceptions of their treatment at the time. Click here for file

Additional file 3 Example of care plan sheet; it displays the treatment information at that point in time, including remaining symptoms, diagnosis, the patient’s condition, medication, problems at the ward and solutions, activities, and the goal of hospital treatment. Click here for file
