# Supplementary material for: Efficacy of shared decision making on treatment satisfaction for patients with first-admission schizophrenia: study protocol for a randomised controlled trial
Source: BMC Psychiatry. 2014 Apr 14;14:111. doi: 10.1186/1471-244X-14-111 (PMC4021257; doi:10.1186/1471-244X-14-111)
Supplement: Additional file 1 — Parts and timing plot; it is a summary of the study parts and their timing in the intervention and control groups. [file 1471-244X-14-111-S1.doc]

| Time Line | | Intervention Group | Control Group |
| --- | --- | --- | --- |
| Baseline Assessment | |  |  |
| Randomization | |  |  |
| Once a Week Intervention During Hospitalization | |  |  |
| Outcome Measurement At Discharge  (within 7 days before discharge from the ward) | |  |  |
| Follow-up (6 months after discharge) | |  |  |
| 1 | Assessment of the patient`s history of hospitalization at a psychiatric ward by researcher. | | |
| 2 | Brief Psychiatric Rating Scale by a ward psychiatrist. | | |
| 3 | Completion of written informed consent by the patient. Reseacher hands over the form to the patient. | | |
| 4 | Questionnaire completed by the patient to elicit his or her view on ongoing treatment. 6 questions to ask how patients think about the treatment with 5-points Linkert-scale. | | |
| A | Meeting following 4. The patient and at least 3 staffs including primary doctor and nurse overview the questionnaire and share their view and information. Each meeting takes 15-20 minutes. | | |
| B | The patient and staff make a weekly care plan sheet at the end of A. | | |
| 5 | The Client Satisfaction Questionnaire-8 Japanese version | | |
| 6 | Drug Attitude Inventory-10 | | |
| 7 | Assessment of treatment continuation (whether the patient come to an appointment with the psychiatrist within 1 month) by medical records or asking patients by telephone. | | |

2

1

1

3

2

3

4

2

5

6

2

5

6

7

7
