# Supplementary material for: Efficacy of shared decision making on treatment satisfaction for patients with first-admission schizophrenia: study protocol for a randomised controlled trial
Source: BMC Psychiatry. 2014 Apr 14;14:111. doi: 10.1186/1471-244X-14-111 (PMC4021257; doi:10.1186/1471-244X-14-111)
Supplement: Additional file 2 — Questionnaire for the patient; a six-item self-reported questionnaire that assesses patients’ perceptions of their treatment at the time. [file 1471-244X-14-111-S2.pdf]

Questionnaire about treatment

1. Do you think you should have been hospitalized?

1	2	3	4	5
Not at all		Neutral		Very much

2. Is your circumstance in the ward appropriate?

1	2	3	4	5
Not at all		Neutral		Very much

3. Do current medications suit you?

1	2	3	4	5
Not at all		Neutral		Very much

4. How is the relationship between you and your ward staffs?

1	2	3	4	5
Poor		Average		Excellent

5. Have you been given sufficient explanation about the treatment?

1	2	3	4	5
Not at all		Neutral		Very much

6. How are you satisfied with the treatment?

1	2	3	4	5
Not at all		Neutral		Very much

Please feel free to write your comment for the treatment below.
