# Supplementary material for: Efficacy of shared decision making on treatment satisfaction for patients with first-admission schizophrenia: study protocol for a randomised controlled trial
Source: BMC Psychiatry. 2014 Apr 14;14:111. doi: 10.1186/1471-244X-14-111 (PMC4021257; doi:10.1186/1471-244X-14-111)
Supplement: Additional file 3 — Example of care plan sheet; it displays the treatment information at that point in time, including remaining symptoms, diagnosis, the patient’s condition, medication, problems at the ward and solutions, activities, and the goal of hospital treatment. [file 1471-244X-14-111-S3.pdf]

<Name>`s Care Plan Sheet

Date:

Written by:

Present condition	<Patient> Being chased by someone, having sleep problem<br><Psychiatrist>Being too irritated, having difficulties to communicate with others.
Diagnosis	Schizophrenia
Reason of hospitalization	<Patient>Violence toward family member.<br><Psychiatrist>Need of medication.
Medication<br>Effect and side-effect	Olanzapin 20mg<br>Effect: Cancel confusion of thoughts.<br>Side-effect: Improve appetite.
Legal status	Involuntary hospitalized
Range of activity	You need to stay in the ward to avoid too much stimulation.<br>You may go outside of the ward when you are with the ward staff.
Problems at the ward and solutions	Want more time to go out.<br>➔ Start going out with family.
Goal of hospital treatment	Being able to live home with the family without too much irritation.
